# A Complete Genome Resource and Bio‐Control Activity of Soil‐Isolated *Pseudomonas citronellolis* Strain M03 Against Onion‐Pathogenic *Burkholderia* Species

**DOI:** 10.1002/mbo3.70325

**Published:** 2026-06-15

**Authors:** Kephas Mphande, Michelle Pena MacLellan, Anoop Anand Malik, Bhabesh Dutta

**Affiliations:** ^1^ Department of Plant Pathology University of Georgia Tifton Georgia USA; ^2^ Department of Biology Valdosta State University Valdosta Georgia USA

**Keywords:** biocontrol, *Burkholderia* spp, genome, onion, *Pseudomonas citronellolis*

## Abstract

*Pseudomonas citronellolis* M03 was isolated from the soil of a field, which has been under continuous onion production for approximately 20 years. The soil was naturally infested with onion‐pathogenic *Burkholderia* spp. but over the last 2 years bacterial populations had declined. The genome of the bacterial isolate was sequenced. The average nucleotide identity (ANIm) analysis showed > 97% identity and > 84% alignment coverage with the type strain *P. citronellolis* DSM50332^T^. Digital DNA‐DNA hybridization (dDDH) values were 73.6% (formula d4) and 77.8% (formula d0), exceeding the 70% species delineation threshold. The genome comprised of a single 6.73 Mb circular chromosome encoding 5802 coding sequences, including 10 pseudogenes, 428 hypothetical genes, and 81 RNA genes (15 rRNAs, 66 tRNAs). We also identified two NRP‐metallophore/NRPS biosynthetic gene clusters showing high similarity to the pyoverdine and enantio‐pyochelin clusters of *Pseudomonas protegens* Pf‐5, suggesting the presence of uncharacterized metabolites with potential ecological or biocontrol functions. Phenotypic assays demonstrated strong antagonistic activity of *P. citronellolis* M03 against *Burkholderia cepacia* and *Burkholderia gladioli in vitro*, reducing their populations by > 10‐fold and approximately 50%, respectively, after 48 h of co‐inoculation. Assessment of onion bulb rot showed significant reductions in both disease incidence and severity when *P. citronellolis* M03 was co‐inoculated with *B. cepacia*. The ability of *P. citronellolis* M03 to persist and exert antagonistic effects in complex soil microflora highlights its ecological fitness and adaptability in natural environments. This study provides a valuable genomic resource and experimental evidence for the biocontrol capability of this potentially beneficial bacterium.

## Graphical abstract

*Pseudomonas citronellolis* M03, isolated from field soil, was sequenced and its genome revealed ten biosynthetic clusters, including siderophore NRPS. Phenotypic assays showed strong antagonism against onion‐pathogenic *Burkholderia* spp., reducing *B. cepacia* populations by > 10‐fold and lowering sour skin severity under greenhouse conditions, highlighting its potential in integrated disease management.

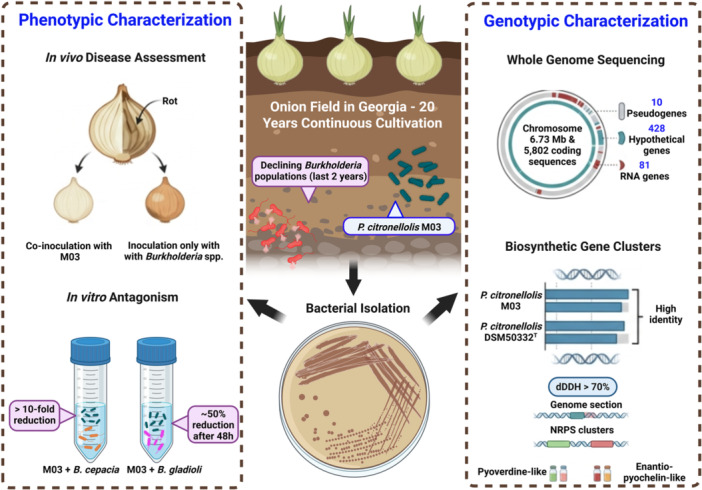

## 1 Introduction

*Pseudomonas citronellolis* belongs to the genus *Pseudomonas* of the family Pseudomonadaceae, which comprises of a large group of bacteria that are ecologically and functionally diverse (Sneath et al. 1980). To date, more than 300 *Pseudomonas* species have been identified, representing more than two‐thirds of all Pseudomonadaceae members (Rudra and Gupta 2025). Some *Pseudomonas* species are pathogenic to humans and plants (Peix et al. 2009; Rudra and Gupta 2025), but many are beneficial. These include strains with strong catabolic abilities for degrading monoterpenes and hydrocarbons, potentially useful in bioremediation (Bhattacharya et al. 2003; Remus‐Emsermann et al. 2016; Adhikary et al. 2019), as well as plant growth‐promoting species that synthesize growth‐stimulating plant hormones and biocontrol species that suppress deleterious microorganisms through growth antagonism (Jousset et al. 2006; Anderson and Kim 2018; Mullins et al. 2019; Sun et al. 2023; Willis et al. 2025). For instance, *P. citronellolis* enhanced chickpea growth and biomass under high arsenic stress, demonstrating potential for bioremediation and reclamation of contaminated or marginal soils (Adhikary et al. 2019). In a separate study, *P. citronellolis* thrived on the phyllosphere, a waxy leaf surface rich in long‐chain aliphatic compounds, possibly breaking down and utilizing these compounds as nutrients. Additionally, *P. citronellolis* was found to harbor genes for indole‐3‐acetic acid biosynthesis (Bhattacharya et al. 2003; Remus‐Emsermann et al. 2016; Adhikary et al. 2019), indicating a role in promoting plant growth (Remus‐Emsermann et al. 2016). However, its potential as a biocontrol agent remains largely unexplored.

Sour skin and slippery skin of onion (*Allium cepa*), caused by the soil‐borne bacteria *Burkholderia cepacia* and *Burkholderia gladioli* pv. *allicola*, respectively, are major threats to onion production worldwide (Jacobs et al. 2008). These bulb‐rotting diseases result in significant post‐harvest losses, and effective control measures remain limited (Gonzalez et al. 1997; Deborah and Mark 2023; Paudel et al. 2024b, 2024a). The antagonistic potential of soil microflora against plant pathogens has been widely recognized, yet this resource remains underexploited for managing onion bulb rot (Chandrashekara et al. 2012; Schlatter et al. 2017; Jayaraman et al. 2021; Sagova‐Mareckova et al. 2023; Todorović et al. 2023; Harmsen et al. 2024; Priyadarshini et al. 2025; Willis et al. 2025). In particular, *Pseudomonas* species are prolific producers of secondary metabolites with potent antimicrobial activity (Leisinger and Margraff 1979; Jousset et al. 2006; Gross and Loper 2009; Sonnleitner and Haas 2011; Shahid et al. 2018; Willis et al. 2025), making them attractive biocontrol candidates (Alattas et al. 2024).

Several *Pseudomonas‐*based products are already commercialized as biopesticides, including Cedomon (*Pseudomonas chlororaphis* MA342), Zequanox (*Pseudomonas fluorescens* strain CL145A), BioJect (*P. chlororaphis* strain Tx‐1), and Bio‐save (*Pseudomonas syringae* strain ESC‐10) (Whitledge et al. 2015; Anderson and Kim 2018; Hernandez‐Jerez et al. 2020; Sun et al. 2023). Moreover, an increasing number of biocontrol candidates within the *Pseudomonas* genus are actively being identified. For instance, *Pseudomonas chlororaphis* subsp. *aurantiaca* strain SRP19 has demonstrated dual functionality as a plant growth‐promoting rhizobacterium and an effective biocontrol agent against Fusarium wilt in sugarcane (Chetan et al. 2024). Similarly, *Pseudomonas poae* JSU‐Y1 exhibited antagonistic activity against *Penicillium expansum*, the causal agent of mold rot in apple fruits (Ren et al. 2021). In hydroponic systems, *Pseudomonas* spp. strain IALR1619 conferred short‐ to medium‐term protection against Pythium blight in cucumber seedlings (Amaradasa et al. 2024). More recently, *Pseudomonas* spp. strain WP18 was reported to possess broad‐spectrum antimicrobial activity against a diverse array of plant‐pathogenic fungi and bacteria (Willis et al. 2025). Although recent efforts have begun to expand genomic data for environmentally beneficial *Pseudomonas* strains, large‐scale genomic analyses remain skewed toward clinical and plant‐pathogenic isolates (Remus‐Emsermann et al. 2016; Willis et al. 2025). This imbalance limits our ability to uncover the molecular and physiological mechanisms that underpin the biocontrol potential of many promising *Pseudomonas* species (Loper et al. 2007; Remus‐Emsermann et al. 2016). In this study, we present a phenotypic characterization and the complete genome assembly of *Pseudomonas citronellolis* strain M03, a promising biocontrol candidate isolated for its antagonistic activity against two plant‐pathogenic *Burkholderia* species within the *B. cepacia* complex. The availability of this genome provides a foundation for investigating the genetic basis of antagonism in *P. citronellolis* and for exploring its potential as a biocontrol agent.

## 2 Materials and Methods

### 2.1 Bacterial Isolations

Strain M03 was isolated from a research field at the Blackshank farm, University of Georgia, Tifton, Georgia, USA, where onion was grown as a monocrop every year for the previous 20 years. We observed a relatively low recovery of *Burkholderia* species from soil samples from this field on a *B. cepacia*‐selective agar (BCSA) (Henry et al. 1997; Yu et al. 2025) compared to other fields that were not previously planted with onions (data not shown). Briefly, 1 g of soil was dissolved in 10 mL of 0.01 M phosphate buffered saline (PBS) (1.7 mM KH_2_PO_4_, 10 mM Na_2_HPO_4_, 2.7 mM KCl, and 136 mM NaCl, pH 7.4) and serially diluted in PBS to 10^−5^ and a 100 µL of aliquot from each dilution was plated on BCSA plates amended with cycloheximide (100 μg/mL). Plates were incubated at 37°C for 48 h. The resultant colonies were re‐streaked and purified for further identification. The selected colonies were characterized by PCR amplifying the 16S rRNA gene using primers 27 F and 1492 R (Frank et al. 2008). The resultant products were Sanger sequenced by Eurofins genomics (Louisville, KY). Using BLAST default settings, the amplicon sequences were searched against the National Center for Biotechnology Information (NCBI) database for preliminary identification of the organism. The selected colonies were stored at −80°C in 20% glycerol stock and a copy was sent for the whole genome sequencing.

### 2.2 Whole Genome Sequencing, Assembly, and Annotation

Genomic DNA extraction, sequencing, read assembly, and assembly quality analyses of strain M03 were performed by Plasmidsaurus (Eugene, OR, USA). Briefly, 1 × 10^9^ CFUs from a culture derived from a single colony grown in Luria‐Bertani broth were pelleted by centrifugation (13,000 rpm, 3 min) and washed in 1 mL PBS before being resuspended in 0.5 mL Zymo 1X DNA/RNA Shield (Zymo Research: Irvine, CA, USA) and shipped to Plasmidsaurus at room temperature. A sequencing library was prepared using the Ligation Sequencing Kit V14 and sequenced on a MinION R10.4.1 flow cell. In addition to the long‐read whole‐genome sequencing and assembly analyses, a supplementary check for potential plasmid presence was performed by plasmid DNA extraction using the Monarch Plasmid Miniprep Kit (New England Biolabs, Ipswich, MA, USA) according to the manufacturer's instructions. Extracted DNA was visualized on a 1.0% (w/v) agarose gel (Remus‐Emsermann et al. 2016). Since the Monarch Plasmid Miniprep Kit is optimized primarily for routine recovery of smaller plasmids, this method was used only as a supplementary assessment alongside long‐read whole‐genome sequencing and assembly graph analyses.

Raw FASTQ reads were quality‑filtered by removing the bottom 5% lowest‑quality reads using Filtlong v0.2.1. To generate an initial assembly draft, reads were downsampled to approximately 250 Mb and assembled with Miniasm v0.3 (Li 2016). The downsampled reads were *de novo* assembled by Plasmidsaurus using Flye v2.9.1 (Kolmogorov et al. 2019) with ONT‑specific parameters. Assembly quality control, contiguity, and structural assessment were performed using Bandage v0.8.1 (Wick et al. 2015; Liu et al. 2025). Genome completeness and contamination were evaluated using CheckM v1.2.2 (Parks et al. 2015). Gene prediction and functional annotation were carried out using Bakta v1.6.1 (Tatusova et al. 2016).

The genome was annotated using Prokka v1.14.5 (Seemann 2014) for downstream phylogenomic analyses. A core‑genome alignment was generated using Roary v3.13.0 (Page et al. 2015), and a maximum‑likelihood phylogeny was constructed using RAxML v12.2.0 (Stamatakis 2014) under the GTR + GAMMA substitution model with 1,000 bootstrap iterations. The resulting tree was visualized using the R package ggtree (Yu et al. 2017). Average nucleotide identity (ANI) was calculated using pyani v0.2.13.1 (Pritchard et al. 2016), and digital DNA‐DNA hybridization (dDDH) analyses were performed using the Type Strain Genome Server (TYGS) (Meier‐Kolthoff and Göker 2019).

### 2.3 Sequence Typing for Secondary Metabolites

Biosynthetic gene clusters (BGCs) were identified using antiSMASH v7.1.0 (Blin et al. 2023) with default parameters. Predicted clusters were compared against the Minimum Information about a Biosynthetic Gene cluster (MIBiG) reference database (Terlouw et al. 2023) to determine closest known matches and percent similarity. Cluster boundaries, predicted metabolite classes, and homology scores were extracted from antiSMASH output files for downstream characterization.

### 2.4 In Vitro Phenotypic Characterization

#### 2.4.1 Spot‐On‐The‐Lawn Assay

The spot‐on‐the‐lawn (SOTL) assay (Ribeiro et al. 2024) was used with modifications to evaluate the biocontrol activity of *P. citronellolis* M03 against *B. cepacia* and *B. gladioli* pv. *allicola*. Briefly, liquid overnight cultures of *P. citronellolis* M03, *B. cepacia* ATCC 25416, and *B. gladioli* pv. *allicola* 20GA0385 were prepared by inoculating single colonies into Luria‐Bertani broth and incubated overnight at 37°C in a rotary shaker (MaxQ 4450, Thermo Fisher Scientific; Waltham, MA) at 200 rpm. The cells were harvested by centrifugation at 13,000 rpm (Centrifuge 5430, Eppendorf, Boston, MA) for 1 min, resuspended in 1 mL PBS, and diluted to an optical density (OD) value of 0.2 at 600 nm (~1 × 10^6^ colony forming units (CFU)/mL). Bacterial lawns for *B. cepacia* ATCC 25416, and *B. gladioli* pv. *allicola* 20GA0385 were made on the BCSA and Luria‐Bertani‐agar media, respectively, as previously described (Moran et al. 2016; Ribeiro et al. 2024). In short, aliquots of 100 µl of each strain was pipetted and spread across the plate using glass beads (7‐mm diameter). The culture of *P. citronellolis* M03 was further diluted in 1 mL PBS to an OD of 0.1 at 600 nm (~1 × 10^5^ CFU/mL), and 5 µl of the cell suspension was spot plated onto the lawns of both *Burkholderia* species. At equidistant from the edge of the plate, another spot was made by pipetting 5 µL of kanamycin (Km) (50 µg/mL) as a positive control. The plates were incubated at 37°C for 48 h, with observations made every 24 h, and antimicrobial activity was quantified by measuring the zone of inhibition (*A* =* πr* ^2^) (Ribeiro et al. 2024). For strain M03, the ZOI was determined by subtracting the area occupied by the colony from the total inhibition zone (Ribeiro et al. 2024).

#### 2.4.2 Competitive Exclusion Assay

In the competitive exclusion (CE) assay the previously described protocol (Wagner et al. 2002; Siedler et al. 2020; Kjeldgaard et al. 2022; Wockenfuss et al. 2024; Zhou et al. 2024), was used with some modifications. In brief, 5 µl of each *Burkholderia* species (*B. cepacia* ATCC 25416, and *B. gladioli* pv. *allicola* 20GA0385) was co‐inoculated with 5 µL of *P. citronellolis* M03 at equal concentrations (~1 × 10^5^ CFU/mL each) into 5 mL Luria‐Bertani broth and grown at 37°C with continuous agitation (Centrifuge 5430). A sample of the co‐culture was taken at 24 h and 48 h and bacterial suspensions were tenfold serially diluted in 0.01 M PBS to 10^−5^, of which 100 µL was spread‐plated on to both BCSA and Luria‐Bertani‐agar plates. These plates were subsequently incubated at 37°C for 48 h followed by bacterial enumeration (CFU/mlL).

### 2.5 Whole Onion Bulb Assay

The whole onion bulb assay was performed according to a previously described protocol (Schroeder et al. 2010; Koirala et al. 2021), with modifications. Briefly, onion bulbs (~150 g each; cv. Vidora) were cleaned by removing the outer tunic layers and surface‐sterilized with 70% ethanol. Bacterial cultures were prepared as above and standardized to 0.2 optical density at 600 nm (~1 × 10^6^ CFU/mL). Four treatments that were used in the assay included onion bulbs inoculated with *B. cepacia* ATCC 25416 only, *P. citronellolis* M03 only, onion bulbs inoculated with co‐culture of *B. cepacia* ATCC 25416 and *P. citronellolis* M03, and onion bulbs inoculated with PBS (control). Each treatment had 10 replications (*n* = 10). Onion bulbs were inoculated by injecting 500 μL of the inoculum into the shoulder of each bulb at 45° angle (Schroeder et al. 2010; Koirala et al. 2021), using a sterile needle (26 gauge, 10 mm long, 0.4 mm outer diameter) (BD, Franklin Lakes, NJ, USA), maintaining a 2 cm uniform depth. Onion bulbs were incubated at 28°C for 7 days before evaluating for sour skin symptoms. The necrotic lesion area (*A* = *πr* ^2^) was calculated by measuring the diameter (average of longitudinal and latitudinal diameter). Disease incidence was noted as presence or absence of rotting symptoms, and the disease severity was measured by counting the number of rotten scales. To evaluate the presence of *B. cepacia* ATCC 25416 in the symptomatic onion bulb tissues, 500 mg of tissue from each onion was surface sterilized with 70% ethanol, macerated in a 2 mL microfuge tube containing 1 mL of PBS and plated on to Luria‐Bertani agar medium and incubated at 37°C for 48 h. The presumptive colonies were screened using the *recA‐based* PCR assay (Mahenthiralingam et al. 2000; Payne et al. 2005; Jacobs et al. 2008; Ginther et al. 2015). The PCR reactions consisted of 12.5 μL of 2× GoTaq Green PCR Master Mix (Qiagen), 7.5 μL of sterile deionized water, 2 μlL of template DNA (> 100 ng), and 2 μL (10 mM) of each primer (BUR3 and/or BUR5) (Ginther et al. 2015). The thermocycler settings were 2 min at 95°C, 35 cycles of 30 s at 95°C, 30 s at 59°C, and 30 s at 72°C, and a final cycle of 5 min at 72°C (Ginther et al. 2015). A sample was considered positive for *Burkholderia* spp. if a 376‐bp PCR product was observed upon gel electrophoresis.

### 2.6 Greenhouse Biocontrol Screening

To assess the biocontrol potential of *P. citronellolis* M03 under greenhouse conditions, three treatments were established in 10‐cm‐diameter high density polyethylene plastic pots (Grower's Solution, Cookeville, TN, USA) containing ~1 kg of natural soil. The soil series used was loamy sand, collected from the Hort Hill farm, University of Georgia, Tifton, Georgia, USA. Preliminary soil analysis revealed a low population density of *Burkholderia* spp. of less than 100 CFU per gram of soil (data not shown). Treatments included: Soil inoculated with *B. cepacia* ATCC 25416, soil co‐inoculated with *B. cepacia* and *P. citronellolis* M03, and a PBS control. There were ten replications per treatment. Pots were drenched with 10 mL of bacterial suspension either alone or co‐inoculated with bacterial species containing 1 × 10^8^ CFU/mL of populations based on the individual treatments. Inoculated pots were maintained at 28°C for 14 days, and were irrigated with sterile water at a 2‐day interval thoughout the life of the experiment (Güney and Keske 2025). Pots drenched with 10 mL of PBS served as a control. After incubation, surface‐sterilized onion bulbs (~150 g each;cv. Vidora) were wounded (2 cm deep) at the shoulder at 45° angle using the proximal wide end of a 1 mL pipette tip (Thermo Fisher Scientific, Waltham, MA, USA) and were inoculated with 1 g of soil from the corresponding pots. Bulbs were incubated under the same conditions as the pots for 7 days, and sour skin symptoms were recorded as disease incidence (presence/absence) and severity (number of symptomatic scales). To confirm pathogen presence, 1 g of soil and 500 mg of onion tissue from each replicate were processed for bacterial isolations and detection using *Burkholderia*‐specific *recA‐*PCR assay (Mahenthiralingam et al. 2000; Payne et al. 2005; Jacobs et al. 2008; Ginther et al. 2015). Soil samples were serially diluted (10^−5^) and an aliquot of 100 μL was spread‐plated on BCSA medium. After a period of incubation, presumptive colonies were enumerated, and further selected and screened for Burkholderia spp. identity as described above. Furthermore, for detection of *Burkholderia* spp. from onion bulbs, 500 mg of onion tissue was surface sterilized with 70% ethanol, macerated and spread‐plated on to Luria‐Bertani agar medium followed by *recA*‐based PCR confirmation for *Burkholderi*a spp. identity.

### 2.7 Statistical Analysis

All statistical tests were performed using GraphPad Prism v10.3.0 (GraphPad Software, San Diego, CA, USA). Disease incidence data were analyzed using Fisher's exact test. All other measurements that were continuous data were analyzed using analysis of variance. Fisher's least significant difference (LSD) post‐hoc test (*p* < 0.05) was used for a pairwise comparison. However, multiple mean comparisons were done using Tukey's honestly significant difference with multiple comparisons adjustment at *p* < 0.05.

## 3 Results

### 3.1 Genome Assembly and Annotation

**Figure 1:**
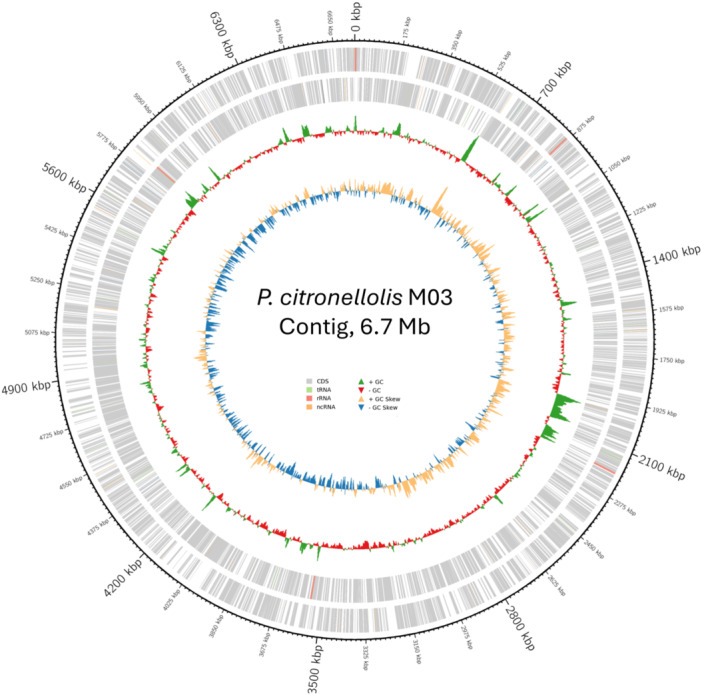
Circular map of the *Pseudomonas citronellolis* M03 genome (6.7 Mb) generated by bakta v1.6.1. Starting from the outermost circle and moving inward, the following features are shown: (1) predicted protein‐coding sequences (CDS) on the forward and reverse strands (gray), (2) tRNA (green), rRNA (orange), and ncRNA (yellow) features, (3) GC content, showing regions above and below the mean GC percentage in blue and orange, respectively, and (4) GC skew, with positive and negative values shown in green and red. Genome coordinates are indicated on the outer scale in kilobase pairs (kbp).

The sequencing run generated 118,670 reads, with a total yield of 825,718,994 bp and an N50 read length of ~12 kb. The longest read was 84,779 bp, and the raw coverage was estimated at 122×, with 101× coverage used for assembly (Table 1). Genome quality was assessed using CheckM v1.2.2 (Parks et al. 2015), which estimated the genome to be 100% complete with 1.9% contamination (Parks et al. 2015; Chklovski et al. 2023), confirming high assembly integrity and low redundancy. The visualization of the GC‐content‐normalized circular genome showed a well‐structured chromosome with clearly delineated coding regions, GC skew, and coverage profiles (Figure 1). The complete genome for strain M03 was deposited in GenBank under the accession number JBRBCS000000000. Contig analysis and assembly graph visualization performed using Bandage (v0.8.1) (Wick et al. 2015; Liu et al. 2025) revealed a single circular chromosome with no additional extrachromosomal contigs, supporting the absence of plasmids in the final long‐read assembly. As a supplementary assessment, plasmid extraction followed by agarose electrophoresis did not reveal detectable plasmid DNA. However, since the extraction method is optimized mainly for smaller plasmids, plasmid absence was inferred primarily from the long‐read whole‐genome assembly and assembly graph visualization. Automated genome annotation (Bakta v1.6.1) provided by Plasmidsaurus identified 5939 genes, including protein‐coding sequences, rRNAs, and tRNAs (Figure 1) (Tatusova et al. 2016).

**Table 1:** Analysis summary of the genome assembly for *Pseudomonas citronellolis* M03, isolated from onion field soil from Tifton, GA, United States.

Genome Feature	*P. citronellolis* M03
Total bp sequenced	825,718,994
Total number of reads	118,670
Longest read (bp)	84,779
N50 (bp)	12,834
Assembly software	Flye v2.9.1
Sequencing technology	Oxford Nanopore (ONT)
Estimated raw coverage	122x
Number of contigs	1
Assembled coverage	101x
Genome size (Mb)	6.7
GC content (%)	67.5
Number of genes annotated	5939
tRNAs	66
rRNAs	15
ncRNA	55

### 3.2 Taxonomic Affiliation

The taxonomic affiliation of M03 was initially determined as *P. citronelollis* based on the 16S rRNA gene analysis. To further validate species‐level classification, average nucleotide identity (ANI) was calculated using pyani v.0.2.13.1 (Pritchard et al. 2016). The comparison was conducted with closely related Pseudomonas genomes, which indicated that *P. citronellolis* M03 shared > 97% identity and > 84% alignment coverage with the type strain *P. citronellolis* DSM50332^T^ (Table SI1a, SI1b and Figure SI1), meeting the accepted species boundary threshold of ≥ 95% based on ANI (Richter and Rosselló‐Móra 2009). Species‐level classification was confirmed by digital DNA‐DNA hybridization (dDDH) analysis using the Type (Strain) Genome Server (TYGS) platform (Meier‐Kolthoff and Göker 2019). *P. citronellolis* M03 exhibited dDDH values of 73.6% (formula d4) and 77.8% (formula d0) relative to *P. citronellolis* LMG 18378 ^T^ (Table SI2), exceeding the 70% dDDH threshold typically used for species delineation (Meier‐Kolthoff et al. 2013). The genome GC content of 67.5% for *P. citronellolis* M03 was also consistent with reference *P. citronellolis* genomes.

**Figure 2:**
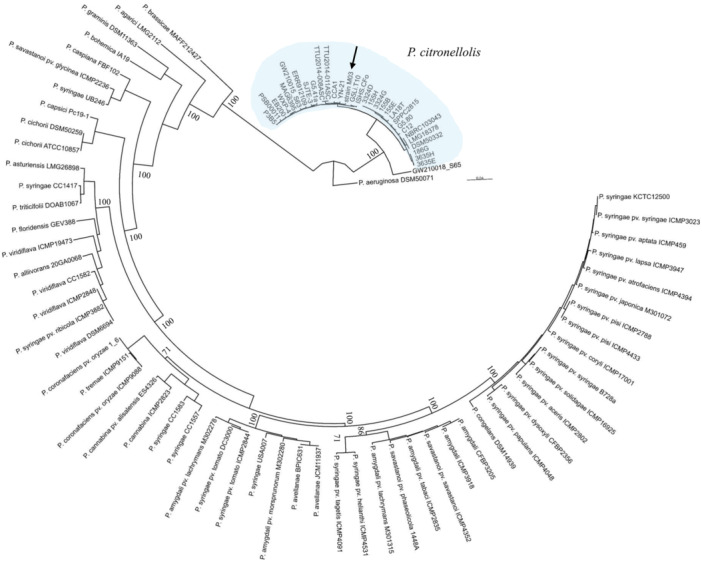
Maximum likelihood phylogeny based on core‐genome of 90 *Pseudomonas* strains. The phylogenetic tree was inferred using RAxMLv12.2.0 under the GTR + GAMMA substitution model with 1000 bootstraps and visualized with R package ggtree. *P. citronellolis* M03 is indicated with a black arrow. Bootstrap values are shown along the branches of the tree. *P. aeruginosa* was used as an outgroup.

For phylogenetic analysis, the genome assembly of *P. citronellolis* M03 was annotated using Prokka v1.14.5 (Seemann 2014), and analyzed with Roary v3.13.0 (Page et al. 2015) to generate a core‐genome alignment. A maximum‐likelihood phylogeny was inferred with RAxMLv12.2.0 (Stamatakis 2014) under the GTR + GAMMA substitution model and 1,000 bootstraps replicates. The analysis included 58 type/representative *Pseudomonas* species and 31 *P. citronellolis* genomes retrieved from NCBI RefSeq to represent phylogenetic diversity within the genus and to provide context for species‐level placement of *P. citronellolis* M03. *Pseudomonas aeruginosa* DSM 50071 was used as the outgroup. The final cladogram was visualized with the R package ggtree (Yu et al. 2017). *P. citronellolis* M03 clustered with other *P. citronellolis* strains in a monophyletic clade (Figure 2). These complementary approaches provide robust evidence that strain M03 is a member of the *P. citronellolis* species. Interestingly, *P. citronellolis* GW210018_S65 did not cluster with the other *P. citronellolis* strains and showed an ANI of > 91% identity with the type strain *P. citronellolis* DSM50332^T^, indicating that strain GW210018_S65 may represent a new species.

### 3.3 Secondary Metabolite Biosynthetic Gene Clusters Identified in *P. citronellolis* M03

To determine if the genome of *P. citronellolis* M03 harbors biosynthetic gene clusters associated with secondary metabolite or antibiotic production, we analyzed the assembled genome using antiSMASH 7.1.0 (Blin et al. 2023). The analysis predicted ten distinct biosynthetic gene clusters (Table 2), several of which share high similarity with characterized clusters in the MIBiG database (Terlouw et al. 2023). Two noteworthy clusters were classified as NRP‐metallophore_NRPS and displayed high similarity to pyoverdine and enantio‐pyochelin biosynthetic clusters from *Pseudomonas protegens* Pf‐5. Specifically, Region 1.7 (3,157,453−3,260,870 bp) showed 98% similarity to the Pf‐5 pyoverdine gene cluster (Stintzi et al. 1999), while Region 1.8 (3,277,256−3,328,387 bp) exhibited 100% similarity to the enantio‐pyochelin cluster (Youard et al. 2007). These siderophore‐associated gene clusters are known to contribute to iron acquisition and may also be involved in antagonistic interactions with plant pathogens. Additionally, Region 1.1 (896,496−917,383 bp) showed 77% similarity to the 12‐epi‐hapalindole C isonitrile biosynthetic cluster from *Hapalosiphon welwitschii* UTEX B1830, a cyanobacterial strain known for producing antimicrobial indole alkaloids (Hillwig et al. 2014). Region 1.4 (2,588,624−2,610,765 bp) matched the lankacidin C biosynthetic cluster from *Streptomyces rochei*, although with a lower similarity of 37% (Suwa et al. 2000). Both clusters may represent novel or divergent natural product pathways.

**Table 2:** Predicted biosynthetic gene clusters (BGCs) in the genome of *Pseudomonas citronellolis* M03, as identified by antiSMASH v7.1.0.

Cluster	Type	Position	Most Similar Cluster*	Type of compounds	% similarity**
Region 1.1	Terpene‐precursor	896,496–917,383	N‐acyl lysine biosynthetic gene cluster from *Pseudomonas aeruginosa* LESB58 (Cohen et al. 2017)	Other	77%
Region 1.2	NAGGN	1,681,818–1,696,620	Scytodecamide biosynthetic gene cluster from *Scytonema* sp. UIC 10036 (Crnkovic et al. 2020)	Other	44%
Region 1.3	Betalactone	2,516,484–2,539,153	Mid‐chain acyl sugars biosynthetic gene cluster from *Solanum lycopersicum* (Fan et al. 2020)	Other	48%
Region 1.4	Redox‐cofactor	2,588,624–2,610,765	Belactosin A biosynthetic gene cluster from *Streptomyces sp*. (Wolf et al. 2017)	Other	37%
Region 1.5	RiPP‐like	2,918645–2,929,490	Curacin A biosynthetic gene cluster from *Moorea producens* 19 L (Gu et al. 2009)	Other	37%
Region 1.6	RiPP‐like	3,123,867–3,134,673	Molybdenum cofactor biosynthetic gene cluster from *Rhodobacter capsulatus* (Solomon et al. 1999)	Other	49%
Region 1.7	NRP‐metallophore, NRPS	3,157,453–3,260,870	Pf‐5 pyoverdine biosynthetic gene cluster from *Pseudomonas protegens* Pf‐5 (Stintzi et al. 1999)	NRP	98%
Region 1.8	NRP‐metallophore, NRPS	3,277,256–3,328,387	Enantio‐pyochelin biosynthetic gene cluster from *Pseudomonas protegens* (Youard et al. 2007)	NRP	100%
Region 1.9	Ranthipeptide	3,399,121–3,420,551	Pf‐5 pyoverdine biosynthetic gene cluster from *Pseudomonas protegens* Pf‐5 (Stintzi et al. 1999)	Other	53%
Region 1.10	Azole‐containing‐RiPP	3,863,008–3,888,593	Choline biosynthetic gene cluster from *Aspergillus nidulans* FGSC A4 (Hai et al. 2019)	Other	53%

*Note:* antiSMASH v7.1.0: A comprehensive platform for the identification and annotation of secondary metabolite biosynthetic gene clusters in microbial genomes (Blin et al. 2023).

Abbreviations: NRP, Non‐ribosomal peptide; RiPP, Ribosomally synthesized and post‐translationally modified peptide; NAGGN, N‐acetylglutaminylglutamine amide‐like; NRPS, Nonribosomal peptide synthetase.

* MIBiG database: The Minimum Information about a Biosynthetic Gene cluster repository containing curated information on experimentally validated BGCs (Terlouw et al. 2023).

** Similarity percentages indicate the amino acid similarity between predicted clusters in M03 and the closest characterized BGCs in MIBiG. High‐confidence predictions typically exceed 70% similarity.

The remaining clusters were classified as RiPP‐like, ranthipeptide, betalactone, redox‐cofactor, NAGGN, and azole‐containing RiPP and showed modest or no homology to known biosynthetic gene clusters in the MIBiG database. These clusters may encode previously uncharacterized metabolites with potential ecological or biocontrol roles. Further biochemical or mutational analyses will be necessary to functionally validate the products of these clusters. This genome resource is a foundation for further investigations into the potential mechanisms of the antimicrobial activity of *P. citronellolis* M03.

### 3.4 *P. citronellolis* M03 Inhibited the Growth of *B. cepacia* ATCC 25416, and *B. gladioli* pv. allicola 20GA0385 In Vitro

**Figure 3:**
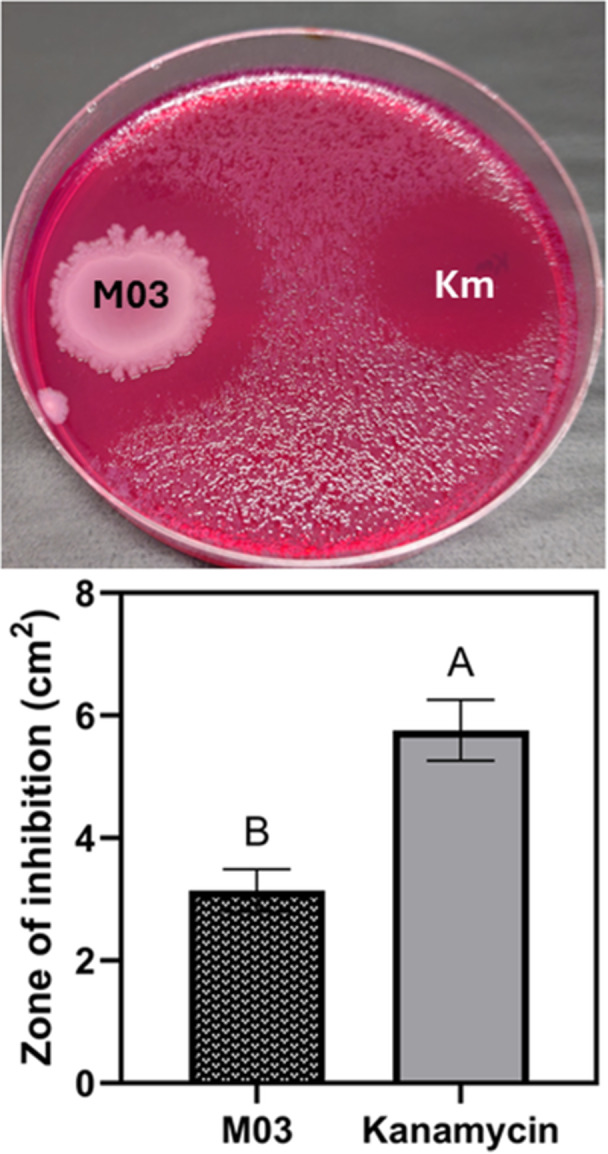
Antimicrobial activity of *Pseudomonas citronellolis* M03 against *Burkholderia cepacia* ATCC 25416. The picture shows the zone of inhibition caused by *P. citronellolis* M03 (left) and kanamycin (km) 50 µg/mLl (right) on the lawn of *B. cepacia* ATCC 25416 after 48 h of incubation on the *B. cepacia* selective agar (BCSA) medium using the spot‐on‐the‐lawn (SOTL) assay. The ZOI caused by *P. citronellolis* M03 was estimated after 48 h of incubation by measuring the clearing zone outside the growing point of inoculation (total area minus area covered by strain M03). Three biological replicates and five technical replications were used. The bar graphs indicate the mean ± standard error of the mean (*n* = 15 per treatment), and bars followed by the same letter were not statistically different (*p* < 0.05). ANOVA was performed using Fisher's Least Significant Difference (LSD) test. Data are representative of two independent experiments.

**Figure 4:**
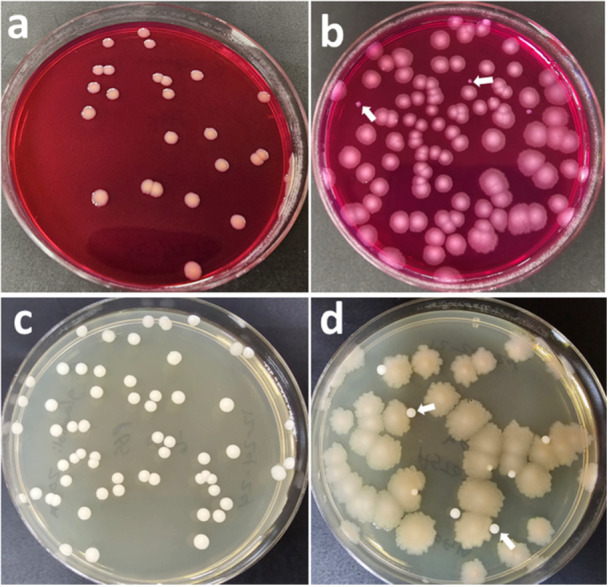
Growth inhibition of *Burkholderia cepacia* ATCC 25416, and *Burkholderia gladioli* pv. *allicola* 20GA0385 when co‐cultured with *Pseudomonas citronellolis* M03. Pictures show the growth inhibition of *B. cepacia* ATCC 25416 (top) and *B. gladioli* pv. *allicola* 20GA0385 (bottom) after 48 h of co‐culturing in Luria Bertani broth followed by spread‐plating of a 100 µL aliquot from a ten‐fold serial dilution (10^−5^) of the culture on *B. cepacia* selective agar (BCSA) and Luria‐Bertani agar media, respectively. The panels indicate; a: *B. cepacia* ATCC 25416 colonies on BCSA medium resulting from the spread‐plating of bacterial suspension incubated for 48 h in Luria‐Bertani broth (control); b: Dominant colonies of *P. citronellolis* M03 (larger, flat colonies with irregular margins) on BCSA medium when bacterial suspension of co‐cultured strains (*B. cepacia* ATCC 25416 and *P. citronellolis* M03) were spread‐plated after 48 h of incubation in Luria‐Bertani broth; c: *B. gladioli* pv. *allicola* 20GA0385 colonies on Luria‐Bertani agar medium resulting from the spread‐plating of bacterial suspension incubated for 48 h in Luria‐Bertani broth (control); d: Dominant colonies of *P. citronellolis* M03 (larger, flat colonies with irregular margins) on Luria‐Bertani agar medium when bacterial suspension of co‐cultured strains (*B. gladioli* pv. *allicola* 20GA0385 and *P. citronellolis* M03) were spread‐plated after 48 h of incubation in Luria‐Bertani broth. The white arrows show the *B. cepacia* ATCC 25416 and *B. gladioli* pv. *allicola* 20GA0385 colonies.

**Figure 5:**
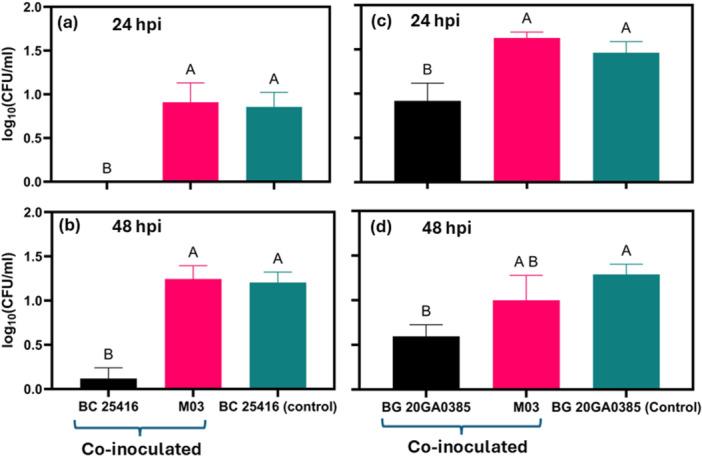
Growth inhibition of *Burkholderia cepacia* ATCC 25416 and *Burkholderia gladioli* pv. *allicola* 20GA0385 by *Pseudomonas citronellolis* M03. Both *Burkholderia* species were co‐cultured with strain M03 in Luria‐Bertani broth, followed by plating 100 µL aliquots of 10^−5^ serial dilutions onto BCSA (for *B. cepacia*) and Luria‐Bertani agar (for *B. gladioli*) media. Panels (a) and (b) indicate inhibition of *B. cepacia* at 24 and 48 h post‐inoculation (hpi), respectively. Panels (c) and (d) indicate inhibition of *B. gladioli* at 24 and 48 h hpi, respectively. Each assay included three biological replicates and three technical replicates (*n* = 9). Data are presented as mean ± standard error. Bars sharing the same letter are not significantly different (*p* < 0.05; ANOVA followed by Tukey's HSD test). Results are representative of two independent experiments.

To assess the biocontrol activity of *P. citronellolis* M03, we conducted two *in vitro* assays; the SOTL (Ribeiro et al. 2024) and the CE (Wagner et al. 2002; Siedler et al. 2020; Kjeldgaard et al. 2022; Wockenfuss et al. 2024; Zhou et al. 2024) assays. The SOTL assay demonstrated that *P. citronellolis* M03 inhibited *B. cepacia* ATCC 25416, producing zones of inhibition comparable to that produced by kanamycin (50 µg/ml), although smaller in size (Figure 3). Since *B. gladioli allicola* 20GA0385 was sensitive to gentamicin, an ingredient in BCSA medium, the Luria‐Bertani agar was used for the assays, and interestingly, similar inhibition results were also observed (data not shown). The CE assay showed significant (*p* < 0.05) reduction in colony counts for both tested plant‐pathogenic *Burkholderia* spp., with *B. cepacia* ATCC 25416 exhibiting more than 10‐fold reduction in populations after 48 h of co‐culture. *B. gladioli allicola* 20GA0385 growth was reduced by approximately 50% compared to the control. Both tested *Burkholderia* strains showed reduced growth in the presence of *P. citronellolis* M03 (Figures 4 and 5). Bacterial populations showed a pronounced reduction for all tested *Burkholderia* spp., with *B. cepacia* ATCC 25416 being the most affected at more than 10‐fold reduction (Figure 5). After 48 h, overall bacterial populations declined, but *P. citronellolis* M03 remained dominant in culture.

### 3.5 *P. citronellolis* M03 Significantly Reduced Sour Skin Incidence and Severity in Onion Bulbs Inoculated With Mixed Cultures of Strain M03 and *B. cepacia* ATCC 25416 Under Controlled Conditions

**Figure 6:**
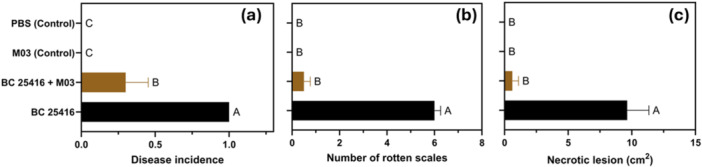
*Pseudomonas citronellolis* M03 significantly reduced sour skin incidence and severity on onion bulbs inoculated with mixed cultures of strain M03 and *B. cepacia* ATCC 25416 in controlled laboratory conditions. Yellow onion (cv. Vidora) bulbs were inoculated with 500 µL inoculum of either 1 × 10^8^ CFU/mL of *B. cepacia* ATCC 25416 (alone), both *B. cepacia* ATCC 25416 and *P. citronellolis* M03 (mixed), *P. citronellolis* M03 (alone), or with 0.01 M phosphate buffered saline (PBS) as control. Sour skin symptoms were evaluated at 7 days post inoculation (DPI). Panels show; (a) sour skin incidence on onion bulbs, (b) disease severity measured by the number of necrotic scales, and (c) the necrotic lesion on the outermost scale of each onion bulb. The bars indicate the mean ± standard error of the mean (*n* = 10), and bars followed by the same letter were not statistically different (*p* < 0.05), ANOVA was performed using Tukey's HSD test. Data are representative of two replicate experiments.

Inoculation of co‐cultures of *P. citronellolis* M03 and *B. cepacia* ATCC 25416 on onion bulbs significantly (*p* < 0.05) reduced sour skin (40%) compared to bulbs inoculated with *B. cepacia* alone (100%) (Figure 6a). Disease severity, measured by the number of rotten scales and necrotic lesion size, showed a similar trend with significantly reduced disease severity in bulbs that were co‐inoculated with *P. citronellolis* M03 and *B. cepacia* ATCC 25416 compared to *B. cepacia* ATCC 25416 alone (Figure 6b,c). The mean number of rotten scales (1.0) and necrotic lesion area (1.5 cm^2^) were significantly lower for the co‐inoculated treatment than the *B. cepacia* ATCC 25416 alone treatment (number of rotten scales = 6; necrotic lesion area 9.5 cm^2^). When *P. citronellolis* M03 was inoculated alone, no rotting symptoms were observed, like in the PBS‐inoculated bulbs (Figure 6 and Figure SI2). *B. cepacia* was recovered from all symptomatic bulbs that displayed sour skin symptoms in either *B. cepacia* ATCC 25416 alone or in the co‐inoculated treatment. In contrast, *P. citronellolis* was not isolated from any symptomatic bulbs, and neither *B. cepacia* nor *P. citronellolis* colonies were recovered from asymptomatic bulbs.

### 3.6 *P. citronellolis* M03 Significantly Reduced *B. cepacia* Populations in Natural Soil and Sour Skin on Onion Bulbs Under Greenhouse Conditions

**Figure 7:**
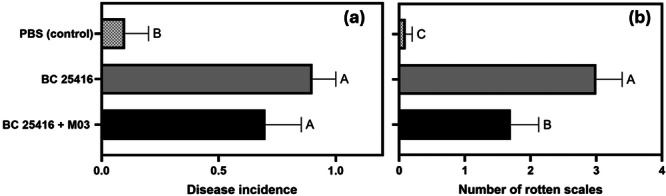
*Pseudomonas citronellolis* M03 significantly reduced disease severity on onion bulbs inoculated with infected soil under greenhouse conditions. Treatments included field soil co‐inoculation of *B. cepacia* and strain M03, *B. cepacia* alone, and phosphate buffered saline (PBS) control. Inoculated pots were kept in the greenhouse for 14 days, thereafter, surface‐sterilized onion bulbs (cv. Vidora) were wounded and inoculated with ~1 g of soil from each treatment. Bulbs were incubated for 7 days at 28°C, and sour skin symptoms were assessed as disease incidence and severity (number of necrotic scales). Panels show: (a) disease incidence and (b) severity. Bars represent mean ± standard error (*n* = 10). Bars sharing the same letter are not significantly different (*p* < 0.05; ANOVA). Data represents two independent experiments.

Under greenhouse conditions, *P. citronellolis* M03 significantly reduced (*p* < 0.05) the populations of *B. cepacia* ATCC 25416 (1 × 10^4^ CFU/g) in natural soil compared to the treatments that were inoculated with only *B. cepacia* ATCC 25416 (1 × 10^7^ CFU/g) (Data not shown). Although the reduction in onion bulb rot incidence (22%) was not statistically significant when *P. citronellolis* M03 was co‐inoculated with *B. cepacia* ATCC 25416 in the soil, there was a significant decrease (43%) in disease severity (Figure 7). Few symptomatic bulbs were observed in PBS controls, possibly from other existing soil opportunistic microbes as no *Burkholderia* species were recovered from these bulbs.

## 4 Discussion

The present study provides a first comprehensive genomic and phenotypic characterization of *P. citronellolis* M03, highlighting its potential as a biocontrol agent against onion‐pathogenic *Burkholderia* species. *P. citronellolis* M03 exhibited strong antagonistic activity against *B. cepacia* ATCC 25416 and *B. gladioli* pv. *allicola in vitro*, as demonstrated by significant growth inhibition in the SOTL and the CE assays. Notably, *B. cepacia*, which is known for its resistance to multiple antibiotics and predatory bacteria (McNeely et al. 2017), showed more than a ten‐fold reduction in growth after 48 h of co‐culture with *P. citronellolis* M03. *P. citronellolis* M03 substantially reduced the growth of *Burkholderia* spp. *in vitro*, although complete inhibition was not observed. Therefore, additional studies are needed to better understand its antagonistic potency and mode of action. However, whether the antagonistic activity of *P. citronellolis* M03 is bactericidal or bacteriostatic remains unclear and warrants further investigation through targeted mechanistic studies. Overall, these findings support the potential of *P. citronellolis* M03 as a promising biocontrol candidate.

The biocontrol potential of *P. citronellolis* M03 was further validated in the whole onion bulb assays under laboratory and greenhouse conditions, where co‐inoculation with *B. cepacia* significantly reduced sour skin incidence and severity compared to bulbs inoculated with the pathogen alone. This reduction in sour skin symptoms under controlled conditions positions *P. citronellolis* M03 as a promising biocontrol agent. In whole onion bulb assays, *B. cepacia* was consistently recovered from symptomatic tissue, whereas *P. citronellolis* M03 was not detected in symptomatic bulbs, and neither of the organisms were recovered from asymptomatic bulbs. These findings suggest that *P. citronellolis* M03 suppressed pathogen proliferation and symptom development without persistent colonization in onion tissue, consistent with extracellular or pre‐emptive mechanisms such as resource competition, antibiosis, and interference with pathogen establishment, common in many Pseudomonads (Shahid et al. 2018; Alattas et al. 2024; Kumar et al. 2025). Under greenhouse conditions, *P. citronellolis* M03 significantly reduced *B. cepacia* populations in natural soil resulting in a significant reduction in onion bulb rot severity. Although disease incidence was not completely eliminated, the observed reduction in severity suggests that *P. citronellolis* M03 can function under field conditions (Remus‐Emsermann et al. 2016; Adhikary et al. 2019; Ren et al. 2021). The ability of *P. citronellolis* M03 to reduce *B. cepacia* populations and maintain antagonistic activity in natural soil suggests ecological adaptability and functionality in complex soil microflora, which are important attributes for successful biocontrol agents (Galli et al. 2024; Kumar et al. 2025). These findings support a model in which *P. citronellolis* M03 leverages efficient exploitation of limiting resources such as iron, and uses secondary metabolites to interfere with pathogen establishment, resulting in suppression of onion‐pathogenic *Burkholderia* spp. in both simplified and complex environments (Alattas et al. 2024). This emphasizes the potential of using *P. citronellolis* M03 as a component of integrated disease management strategies for the onion production systems.

Genomic analysis revealed ten BGCs, including siderophore‐associated clusters closely related to the pyoverdine and enantio‐pyochelin pathways of Pseudomonas protegens Pf‐5 (Stintzi et al. 1999; Youard et al. 2007). These siderophores, which mediate iron acquisition and competitive exclusion (Burbank et al. 2015; Khasheii et al. 2021; Xie et al. 2024; Lan et al. 2025), may contribute to the observed antagonistic activity. Additional BGCs with similarity to antimicrobial alkaloid and polyketide pathways indicate potential for secondary metabolite production, consistent with in vitro inhibition and warranting further characterization. We speculate that the biocontrol activity of *P. citronellolis* M03 is multifaceted, involving siderophore‐mediated iron competition, antibiosis via. secondary metabolites, and ecological competitiveness in soil and co‐culture (Shahid et al. 2018; Galli et al. 2024; Alattas et al. 2024; Kumar et al. 2025). However, functional analysis through targeted mutagenesis and metabolomic analyses is required to validate the genomic and phenotypic evidence.

Our findings expand the ecological and functional importance of *P. citronellolis*, a species previously recognized for hydrocarbon degradation and plant growth promotion under heavy metal stress (Remus‐Emsermann et al. 2016; Adhikary et al. 2019). The discovery of its biocontrol capability against plant‐pathogenic *Burkholderia* spp. addresses a critical gap in onion disease management, where effective control measures for soilborne bacterial diseases remain elusive (Gonzalez et al. 1997; Deborah and Mark 2023; Paudel et al. 2024a, 2024b). The availability of a complete genome for *P. citronellolis* M03 provides a valuable resource for expanded functional genomic studies on beneficial Pseudomonads and the development of next‐generation biopesticides, an area that has been largely overshadowed by work focusing mainly on clinical and plant‐pathogenic isolates (Loper et al. 2007; Remus‐Emsermann et al. 2016; Willis et al. 2025).

## 5 Conclusions

*P. citronellolis* M03 significantly reduced sour skin disease under laboratory and greenhouse conditions and exhibited strong antagonistic activity *in vitro*, supporting its potential as a biocontrol agent. Genome analysis identified siderophore‐associated and antimicrobial biosynthetic clusters likely involved in biocontrol activity. Future research should focus on elucidating molecular mechanisms and characterizing secondary metabolites. Additional priorities include functional genomics, transcriptomics, ecological fitness studies, expanding biocontrol activity spectrum to other soilborne pathogens, and formulation development for practical applications. Collectively, this work advances our understanding of beneficial pseudomonads and opens new avenues for biocontrol‐based disease management strategies.

## Author Contributions

**Kephas Mphande:** conceptualization, investigation, writing – original draft, methodology, validation, visualization, software, formal analysis, data curation. **Michelle Pena MacLellan:** writing – review and editing, visualization, validation, software, formal analysis, data curation. **Anoop Anand Malik:** validation, visualization, writing – review and editing, software, formal analysis, data curation. **Bhabesh Dutta:** data curation, supervision, resources, project administration, writing – review and editing, methodology, conceptualization, funding acquisition, validation, visualization.

## Ethics Statement

The authors have nothing to report.

## Conflicts of Interest

The authors declare no conflicts of interest.

## Supporting information


**Figure SI1:** Average nucleotide identity (ANI)‐based heatmap showing the status of the M03 strain. The intensity of the color indicates the level of identity of all‐versus‐all genomes as depicted by the scale.

**Figure SI2:** *Pseudomonas citronellolis* M03 inoculation did not result in bulb rot symptoms. Yellow onion (Cv. Vidora) bulbs were inoculated with 500 µl inoculum of either 1 × 10^8^ CFUs per mLof *B. cepacia* ATCC 25416, *P. citronellolis* M03, or 0.01 M phosphate buffered saline (PBS). Onion bulb rot symptoms were evaluated at 7 days post inoculation (DPI). Panels show; (a) sour skin incidence on onion bulbs, (b) disease severity measured by the necrotic lesion on the outermost scale of each onion bulb. The bars indicate the mean ± standard error of the mean (n = 15), and bars followed by the same letter were not statistically different (P < 0.05). Data are representative of two replicate experiments.

Supporting File

## Data Availability

The whole genome sequence of *P. citronellolis* M03 has been deposited at the public repository DDBJ/ENA/GenBank under the accession JBRBCS000000000. The version described in this paper is version JBRBCS010000000. The data that support the findings of this study are openly available in DDBJ/ENA/GenBank at https://www.ncbi.nlm.nih.gov/nuccore/, reference number JBRBCS010000000.
