# Supplementary figures and images for: A Complete Genome Resource and Bio‐Control Activity of Soil‐Isolated Pseudomonas citronellolis Strain M03 Against Onion‐Pathogenic Burkholderia Species
Source: Microbiologyopen. 2026 Jun 15;15(3):e70325. doi: 10.1002/mbo3.70325 (PMC13266271; doi:10.1002/mbo3.70325)

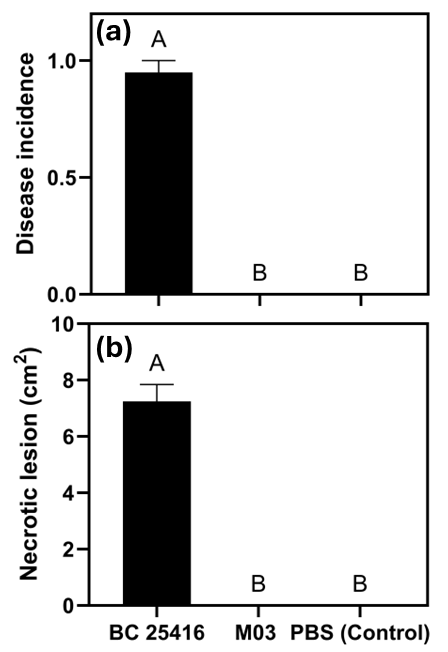

Supplement: Supplementary file 2 — Figure SI2: Pseudomonas citronellolis M03 inoculation did not result in bulb rot symptoms. Yellow onion (Cv. Vidora) bulbs were inoculated with 500 µl inoculum of either 1 × 10^8^ CFUs per mLof B. cepacia ATCC 25416, P. citronellolis M03, or 0.01 M phosphate buffered saline (PBS). Onion bulb rot symptoms were evaluated at 7 days post inoculation (DPI). Panels show; (a) sour skin incidence on onion bulbs, (b) disease severity measured by the necrotic lesion on the outermost scale of each onion bulb. The bars indicate the mean ± standard error of the mean (n = 15), and bars followed by the same letter were not statistically different (P < 0.05). Data are representative of two replicate experiments. [file MBO3-15-e70325-s003.tif]
